# BRON: A blockchained framework for privacy information retrieval in human resource management

**DOI:** 10.1016/j.heliyon.2024.e33393

**Published:** 2024-06-26

**Authors:** Gulshan Kumar, Rahul Saha, Manish Gupta, Tai Hoon Kim

**Affiliations:** a School of Computer Science and Engineering, Lovely Professional University, India; b School of Business, Lovely Professional University, India; c School of Electrical and Computer Engineering, Chonnam National University, South Korea

**Keywords:** Human, Resource, Privacy, Blockchain, Information, Retrieval

## Abstract

The correctness and the true validated data in Human Resource Management (HRM) are important for organizations as the data plays an impactful role in recruiting, developing, and retaining a skilled workforce. On one hand, the validated data in an organization helps in recruiting legitimate skillful employees; on the other hand, keeping the employee's data safe and maintaining privacy laws such as compliance with the General Data Protection Regulation (GDPR) is also an organization's responsibility. Besides, transparency in human resource management operations is crucial because it promotes trust and fairness within an organization. The present HRM systems are centralized in nature and their verifiable credential system is ineffective; this leads to the intentions of internal data sabotage or internal threats. Besides, the organizations' biases also become more prominent.

In this paper, we address the above-mentioned problems with a blockchain framework for HRM to utilize the privacy of data access through a Privacy Information Retrieval (PIR) process. To be specific, our proposed framework called *Blockchained piR of resOurces as humaN (BRON)*, is the first blockchain framework to show an effective mechanism to access data from organizations globally without hampering privacy. BRON uses a generalized user registration process to use the services of data access and in the background, it uses Zero-Knowledge Proofs (ZKPs) for global verification and PIR for privacy-based data retrieval. More specifically, credential verification and ZKP-based PIR are the highlights of our proposed BRON. Another interesting aspect of BRON is the use of Proof-of-Authority (PoA) to validate the anonymity and unlinkability of any HR operation. Finally, BRON has also contributed with a smart contract to incentivize the employees. BRON is very generic and easily be customizable as per the HR requirements. We run a set of experiments on BRON and observe that it is successful in providing privacy-assured data access and decentralized human resource data management. Overall, BRON provides 30% reduced latency and 35% better throughput as compared to the existing blockchain solutions in the direction of HRM.

## 1 Introduction

Blockchain technology is one of the most promising technology enablers in the present world. The transparency, immutability, and security of blockchains offer several benefits that extend far beyond cryptocurrencies like Bitcoin [1]. Blockchain is a transparent and tamper-proof distributed ledger that ensures trust in a trustless environment of decentralization. This makes blockchain an ideal solution for international money transfers, supply chain financing, and digital identity verification [2]. Blockchain has been widely adopted by industries like healthcare by enhancing the security and interoperability of medical records, making patient data more accessible while maintaining privacy [3]. Besides, blockchain has shown a beneficial impact on supply chains; blockchain enables end-to-end visibility, reduces the chances of counterfeiting, and improves traceability. Additionally, blockchain inclusion in voting systems brings transparency and security to electoral processes. Today's digital world not only thinks about the security or confidentiality that is generally obtained through strong cryptographic mechanisms, but is also concerned about privacy, which deals with the data use controls. In this paper, we consider Human Resource Management (HRM) as an application domain of blockchain. The reason for selecting this application domain is the generalization of HRM in almost every industry. Despite the importance of HRM, the decentralized perspective is less researched yet.

Centralized human resource management lacks transparency leading to a biased decision-making process, data manipulation, lack of trust, and falsification [4] [5]. In centralized systems, decision-making processes are often obfuscated giving the space for subjective judgments. This leads to biased hiring, promotion, and compensation decisions. Centralized HRM faces data vulnerability [6] [7]. This can expose sensitive employee information and lead to manipulation or falsification of records, eroding trust in the system. Further, errors in data entry or record-keeping can occur, leading to inaccurate employee profiles or records. These inaccuracies can be exploited or manipulated for personal gain or to disadvantage others. A few individuals or departments often have control over key processes in HRM. This monopoly can result in favoritism, discrimination, or unethical practices. Besides, without a transparent audit trail, it is challenging to hold repudiated parties accountable for their actions. Thus, a lack of accountability is a drawback in a centralized human resource management system [8]. All of these problems in HRM can decrease trust in the organization leading to reduced morale, engagement, and job satisfaction [9] [10].

Blockchain technology addresses many of the above-mentioned issues by offering transparency, security, and decentralized control. Blockchain offers a secure, transparent, and tamper-proof way to store and verify employee data, including qualifications, work history, and performance records. This not only enhances data integrity, but also enhances data control and ownership of individuals. Smart contracts can automate basic HR processes like onboarding, payroll, and performance management; it reduces manual administrative tasks leading to bias-free and fair decision-making. Blockchain's transparency and decentralization also help in reducing discrimination, ensuring fairer hiring and promotion practices. Overall, blockchain proves to be beneficial for HRM by making it more efficient, inclusive, and trustworthy. In the following, we discuss the benefits of blockchain inclusion in human resource management [11] [12]. Blockchain provides a secure and tamper-proof ledger of employee data. This helps in checking the verified qualifications (if a verifiable credential is used separately) or work history, and the feedback or review. Blockchain allows employees to own and control their data leading to effective security measures. Smart contracts in blockchain help to automate various HR processes, such as onboarding, payroll, and benefits; this leads to streamlining operations and avoiding bias or discrimination in decision-making processes and enhances fairer hiring, promotion, and compensation practices. Overall, blockchain technology has the strength to revolutionize HRM, making it more inclusive by ensuring data integrity, reducing bias, enhancing transparency, and giving individuals more control over their personal information. We use Table 1 to summarize the mapping of problems in centralized HRM and solutions by blockchain-based HRM.

**Table 1:** Centralized HRM problems vs solutions by blockchain-based HRM.

Problems in centralized HRM	Solutions by blockchain-based HRM
Sensitive employees' data are stored in a centralized server making it a target of data breaches and cyber-attacks	The decentralized and immutable nature of blockchain ensures the security of the stored data
Verification of credentials, previous work history, or references are influential and complex	Decentralized verifiable credentials ease up the process
Prone to biases in decision-making due to monopoly and centralization	Transparent, consensus-based, and smart contract-based operations avoid biases
Lack of accountability	Each transaction for any operation is accounted transparently
Prone to errors in data entry or record-keeping	Validated and verified through consensus mechanisms.
Increased complexity for multi-stakeholder support	Easy process for multi-stakeholder support using smart contracts

### 1.1 Privacy and zero knowledge proofs (ZKPs) in blockchain

Despite several advantages, blockchain faces privacy issues due to its transparency [13]. In a public blockchain, all transactions are recorded on a distributed ledger and the ledger is accessible by anyone being part of the blockchain. Though this transparency is essential for accountability and trust, it can compromise user privacy. The transaction details are linkable to specific addresses; this can reveal sensitive information about individuals. Private Information Retrieval (PIR) allows users to retrieve information from the blockchain without disclosing their identity or the specific data they are querying, thereby preserving transaction confidentiality and privacy. Apart from financial transactions, blockchains can also store various types of sensitive data, such as personal identifiers, health records, or business contracts. PIR ensures that users access this information securely without exposing it to unauthorized parties. This is critical in permissioned or consortium blockchains used in various sectors such as healthcare, finance, supply chain management, and human resource management, where data privacy regulations are stringent such as General Data Protection Regulations (GDPR) and the Health Insurance Portability and Accountability Act (HIPAA). To apply PIR, we can use various methods such as homomorphic encryption, Zero Knowledge Proofs (ZKPs), Oblivious Transfer (OT), ring signatures, Multi-Party Computation (MPC), proxy re-encryption, differential privacy, and off-chain solutions [14].

Out of the existing methods of PIR in blockchain, we consider ZKP due to its several advantages. ZKPs allow users to prove the validity of a statement without revealing any additional information. This is called selective disclosure property, which is useful in blockchain applications where users may want to prove ownership, authenticity, or compliance with certain conditions without revealing sensitive details. Besides, Modern ZKP constructions, such as Zero-Knowledge Succinct Non-Interactive Argument of Knowledge (zk-SNARKs) and Zero-Knowledge Scalable Transparent ARguments of Knowledge (zk-STARKs) produce minimal computational overhead. The compactness of the proofs also reduces large storage requirements. In ZKPs, anyone can verify the correctness of a proof without a trusted prover leading to decentralized transparency and auditability in blockchain applications [15] [16]. In ZKP, a prover convinces a verifier of the truth of a statement without revealing any additional information. Using cryptographic techniques, the prover generates a proof that demonstrates knowledge of secret information and also preserves privacy. A verifier can verify the proof's correctness without learning anything about the prover's secret; thus, ZKP ensures privacy, confidentiality, and integrity in transactions and computations in blockchains.

### 1.2 Related works

Blockchain has the potential to revolutionize HRM with almost every HR operation being decentralized. However, its adoption in the HRM sector has been relatively slow due to HR professionals' inadequate expertise and/or organizations' resistance to invest in such technological advancements, specifically for HRM operations, and the lack of standardization in blockchain protocols [17]. Moreover, the transparency attribute of blockchains contradicts the privacy requirements of HR data. Finding a balance between transparency and data protection or privacy is a significant challenge for blockchain inclusion in HR. In many countries such as in European Countries, the General Data Protection Regulation (GDPR) has strict regulations regarding employee data and privacy. Complying with these regulations while proceeding with HRM decentralization is difficult. Some companies and startups have started exploring the use of blockchain in HR in the operations of credential verification, recruitment processes, and employee records integrity. For example, SAP uses blockchain for credentials verification [18], Chronobank uses blockchain for decentralized labor exchange [19], and U-Port and Evernym focus on decentralized identity solutions [20].

Despite the huge potential of blockchain inclusion in HR management services, the existing research works fall short in addressing the multi-dimensional HRM operations. A limited number of works show the scope of blockchains in human resource management systems. For example, some recent surveys by R. Ramachandran et al. [21] and P. Chanda et al. [22] show the analysis of the works of blockchain in human resource management. The studies identify some major directions in human resource management, which are profitable with blockchains. The directions include recruitment and talent management, a decentralized hiring process, payroll, and staff engagement skills, and credentials for onboarding and offboarding. Another recent study by B. Godavarthi et al. [23] provides insights on similar facts of employee performance management. Previously, authors have shown a blockchain framework for global human resource data management [24]. Another recent study explores blockchain-based human resource management solutions for addressing current challenges in China's HR management [25]. The system incorporates a robust PBFT consensus algorithm to securely store document data, enhancing traceability and efficiency. Intelligent protocols and IPFS enable local backup and mobile sharing, improving data security and demonstrating advantages in output characteristics, consensus rate, and stability, making it a promising solution for modern HR data protection and sharing needs. As mentioned earlier, the blockchain applications in human resource management are observable in the direction of talent acquisition [26] [27] [28]. Moving ahead, talent uplift and skill development among human resources also show interest in blockchain applications [29] [30].

With the discussion above, it is very clear that the implementation of blockchain applications in the direction of human resource management (in any form) is in the infancy stage. Moreover, the present HRM systems do not employ either the decentralized verifiable credential or privacy-assured data access for human resource data.

### 1.3 Motivation and contribution

The literature study in the direction of blockchain implications in human resources shows that the existing research works and developed frameworks primarily focus on credential verification and talent acquisition. However, they miss out on the point of privacy concerns in all such human resource data for data sharing, data storage, and data access. Let us consider a real-life example. A company XYZ runs with several hundred employees; the company relies on a traditional centralized database system for its human resources data. The database contains sensitive information such as employee salaries, performance reviews, medical records, and personal contact details. Being a centralized system, such HRM becomes a target point of hackers. A cyberattack can reveal all the sensitive HR data leading to identity theft, financial fraud, or other malicious activities. This becomes more dangerous when the attacker is internal. Data breach at Tesla (2023), data exposure at Pegasus Airlines (2022), Cash App's customer data leak by an employee (2022), intellectual property theft by a malicious insider at Yahoo (2022), and stealing of Slack's code repositories due to a compromised vendor (2022) are some of the examples of recent privacy breaches [31]. Besides, multinational organizations such as IBM, Gartner, and MorganPhilips and start-ups such as Etch, Beowulf, Gospel Technologies, Lympo, Peolewave, and WurkNow have adopted blockchain in HRM and also show the problem of credential verification and privacy in HRM [32][33][34][35]. All these motivate to design of a novel blockchain framework to ensure privacy at the time of data sharing in the blockchain and also in the shared data access in HRM. The motivation for using blockchain framework also comes from the advantages provided by this technology. For example, the blockchain's cryptographic algorithms and decentralized architecture make it extremely difficult for attackers to tamper with or gain unauthorized access to the stored HR-related data. Besides, every transaction (data access) on the blockchain can be recorded in a transparent and immutable manner; this enables easy auditing and verification of data integrity. This helps to monitor the internal employees for any fraud or internal attacks. Therefore, we design a blockchain framework for HRM. We call our framework *Blockchained piR of resOurces as humaN (BRON)*. To this end, BRON has the following contributions.

• **Privacy information retrieval**: The existing research works in the direction of decentralized human resource management systems fail to address the privacy issues in data access from blockchain. BRON, our proposed framework, addresses the above-mentioned problem by introducing a proof-based Privacy Information Retrieval (PIR) system.
• **Unlinkability and anonymity**: BRON ensures unlinkability and anonymity, which are the major concerns in blockchain transactions for human resource records. The unlinkability and anonymity assurance is based on the use of encryption-based verifiable credentials and Proof-of-Authority (PoA).
• **Verifiable zero-knowledge proofs**: BRON shows efficient use of verifiable Zero-Knowledge Proofs (ZKPs) by using privacy budgeting and parametric analysis of privacy attributes. To be specific, BRON is the first decentralized HRM framework that shows the benefits of ZKP in PIR.
• **Performance**: We implement BRON on Hypeledger Fabric with HYperledger Caliper and run a set of experiments on BRON. Experimentally, BRON is 30% better in terms of latency and 35% better in terms of throughput.

BRON, our proposed blockchain framework is the first blockchain framework for HRM that uses ZKPs, unlinkability, and anonymity through PoA, and PIR. Consequently, BRON becomes beneficial to handle HR-related data transparently. Besides, the verifiable credentials make it easier to validate the credentials of the employees. With an add-on contribution, BRON helps in privacy-assured data access through PIR; this helps in referential job applications. From the organization's perspective, BRON also helps with auditing services of the HR data; thus, BRON helps in preventing insider attacks or general privacy attacks.

### 1.4 Paper organization

We organize the rest of the paper as follows. Section 2 discusses the proposed framework with detailed functioning of the modules. Section 3 discusses the privacy requirements of our proposed framework. Section 4 shows the performance of the proposed system by analyzing the results. Finally, Section 5 concludes the paper.

## 2 Proposed framework: BRON

In this section, we first describe the research methodology in Section 2.1 followed by a system model in Section 2.2. We discuss the detailed operations in Section 2.4.

### 2.1 Research methodology

We follow a strategic methodology and planning in designing the proposed BRON. We initiate the process by defining the objectives and use cases. The objectives of the proposed BRON are: i) to provide differential privacy in transactions, ii) to ensure privacy while retrieving information from the blockchain, and iii) to automate the process of credential verification and PIR through a smart contract. To accomplish the objectives, we first do the stakeholder analysis and identify three major roles: prover (e.g., employees), verifier (e.g., organization), and issuer/validator (e.g., council, federation, and authoritative entities). At the same time, we also analyze the basic HR operations that need to be in the BRON: registration for both users and companies, verification of credentials, privacy assurance in information retrieval, and smart contact for automating the basic processes. We also include the consent management process and privacy rights obligations in BRON as a part of other services. We then identify the schema for required data related to HRM. We identify publicly available datasets from Kaggle and also generated data out of the transactions. However, this data is hypothetical and does not have a direct connection with any organization; the schema and the features of the dataset are analyzed based on the available HRM datasets. Next, we choose Hyperledger Fabric as a blockchain platform due to its applicability to consortium blockchain. We believe HRM does not have a public blockchain, rather various organizations, councils, and federations can connect to consortium blockchain to use the proposed BRON framework. After selecting the platform, we start designing the algorithms as per the objectives that are of concern. Finally, we code them and integrate them into the Hyperledger Fabric to come up with our proposed BRON.

### 2.2 System model

**Figure 1:**
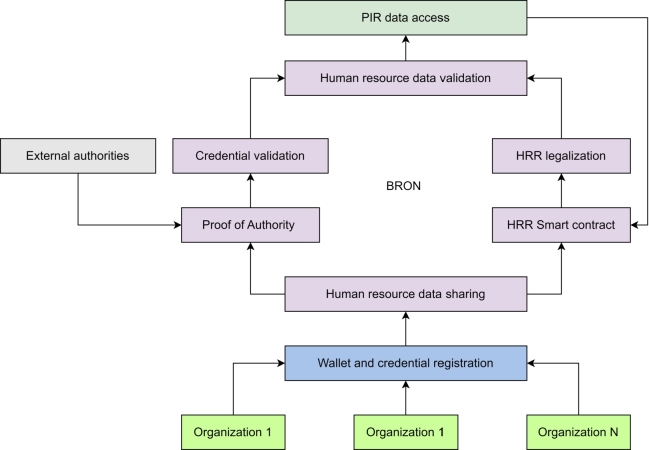
Proposed system model of BRON.

We propose a blockchain framework for privacy-preserved data sharing and access in human resource applications. We call our proposed framework, Blockchained piR of resOurces as humaN (BRON). *BRON* means *source* or *resource* in the Dutch language. BRON is the first blockchain framework to address the issues of likability in human resources data and shows the use of Privacy Information Retrieval (PIR) for stored Human Resource Records (HRRs). Fig. 1 shows a conceptual diagram for the proposed system model of BRON. Through the figure, we show that *N* number of organizations can participate simultaneously in the BRON framework. We consider external authorities such as Certificate Authority (CA) as a separate organization. All the organizations must have credentials (wallets) while the registration process is invoked for a new organization. Any organization is allowed to share data of human resources as a transaction for the blockchain. The transactional data must be validated through Proof of Authority (PoA) consensus before updating in the blockchain. The validation of transaction data ensures the verification of credentials (verifiable credentials or VCs). Smart contracts are optional; however, they provide a smart notion of maintaining human working contracts. These contracts are effective for maintaining employee performance, employee knowledge transfer capability maturation, payroll execution, incentive disbursement, and other organization-based human resource operations. Finally, we use PIR for accessing the data from the blockchain by ensuring privacy attainment.

### 2.3 Consent management and privacy rights

Consent management in HRM is the process of obtaining, recording, and managing consent of the employees in an organization. The task of consent management is executed for various purposes related to data processing and compliance with GDPR or other data protection laws. The consent relates to collecting and processing personal data, sharing information with third parties, conducting background checks, and taxation. Effective consent management ensures individuals' privacy rights and preferences. Another crucial point for HRM is the Right to Access and Data Portability (RADP). RADP allows employees to access and control the data held by employers. Our proposed BRON includes consent management and RADP in the registration process. The consent is also verified at the time of verified credential.

### 2.4 Detailed functions

In this section, we discuss the important functionalities, which are required to understand the overall functioning of our proposed BRON. The functionalities include registration, verifiable credentials, PoA, smart contracts, and privacy information retrieval.

#### 2.4.1 Registration

**Figure 2:**
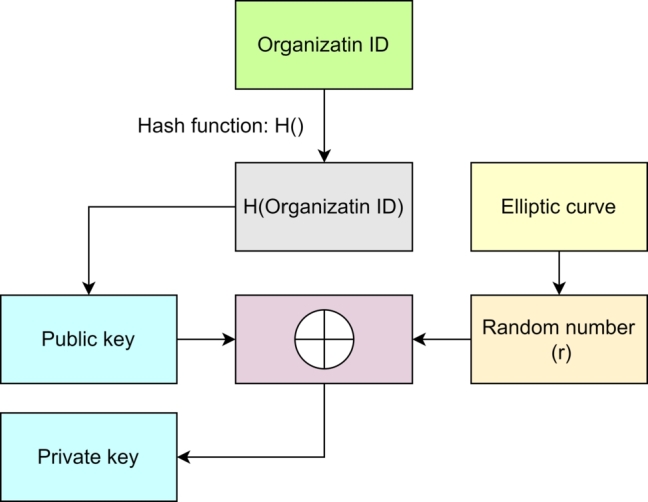
Flowchart for registration in BRON.

BRON uses a simple registration process using elliptic curves. We consider organizations to be a part of a consortium and thus, BRON uses consortium blockchain feasibilities. Following this, an organization willing to participate in the consortium must register itself with the organization ID $\left(O_{ID}\right)$. We keep the scope open to connect the self-sovereign identities for organizations and consider organization-specific identities. Generally, these identities are organization registration numbers affiliated with a governing body of legalization (such as a board, council, or other federations). The registration function of our proposed BRON inputs $\left(O_{ID}\right)$ and outputs public-private key pairs (K+andK−) for each organization willing to participate in the consortium and use the BRON services. Besides, we also use consent management as the individuals can sign the consent with a value of 1. The signature is done by the private key *K*− of the individual participating in the consortium and providing the consent. The registration function creates the hash of $\left(O_{ID}\right)$ with a hash function $H:{\left\{0,1\right\}}^{k}\to {\left\{0,1\right\}}^{k}$; this hash value becomes the wallet address or the identity of an organization in the consortium blockchain and uses the hashed value further in Elliptic Curve Cryptography (ECC) to generate the keys. We use the popular elliptic curve X25519 in the form of $y^{2}=x^{3}+ax+b$. The registration function selects a random point *p* on the elliptic curve with a finite field $Z_{q}$ with order $o=q^{k}$. The relation among *a*, *b*, and *k* must be (a,b)<q. The registration function calculates a random number *r* in the field $Z_{q}⁎$ which is multiplied with the hashed ID to generate the private key for a specific organization. We summarize the process of registration in Algorithm 1 and show a logical flowchart in Fig. 2.

**Algorithm 1:**
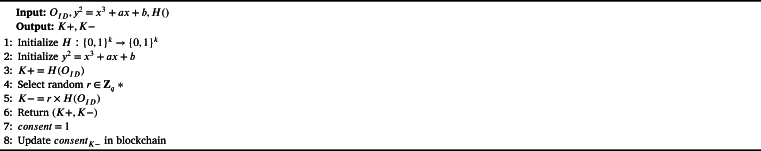
*registration*().

#### 2.4.2 Verifiable credentials

**Figure 3:**
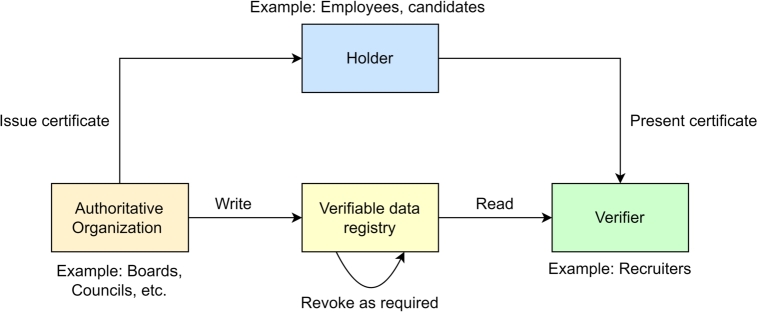
Flowchart for verifiable credential in BRON.

We use the concept of Verifiable Credential (VC) as a provision of employee's and employer's background verification. To achieve this goal, we assume that authoritative organizations such as boards, councils, and other federations are also the part of BRON framework. We provide a logical example to show the usefulness of verifiable credentials in our BRON framework. Let us assume that organization *A* is going to hire an employee *B*, who has produced a qualification certificate of *C*. *A* needs to be confident enough that the certificate *C* produced by *B* is genuine and the signature on *C* is verifiably true. To obtain this perspective, we follow the following process. We consider that a credential or certificate *C* consists of four basic parameters: i) certificate ID $\left(C_{ID}\right)$, ii) issuance timestamp (issuance), iii) expiry timestamp (expiry), and iv) signature of the authority $sign_{auth}$. Note that the validator is similar to the authority. We call the entity to whom the *C* is issued, a holder and the entity where the credential is submitted as the verifier. Therefore, the verifier is applicable to request $\left(v_{req}\right)$ for verification and validation of a *C* whenever required. Thus, *C* becomes *VC*. Note that, consent must be true before the validator is validating the $\left(v_{req}\right)$. The verifier requests to verify *C* with a transaction $v_{req}$ signed by the verifier sign(verifier). The validator (the intended authority) verifies the signature of the $\left(v_{req}\right)$; if the signature is true it checks the values of certificate parameters passed by the verifier and confirms the trueness of *C* along with the timestamp of the verification to ensure the liveliness. If the signature of the requester is not verified, then the verification request is aborted. We show the overall process in Algorithm 2. The decentralization in credential verification serves two purposes: i) once the credential verification is done, the transaction is updated in the blockchain, which remains immutable; thus, repeated verification can be avoided and ii) the validators are also transparent; as a result, frauds in recruitment process can be avoided. We have shown a logical interpretation of the verifiable credential process in Fig. 3.

**Algorithm 2:**
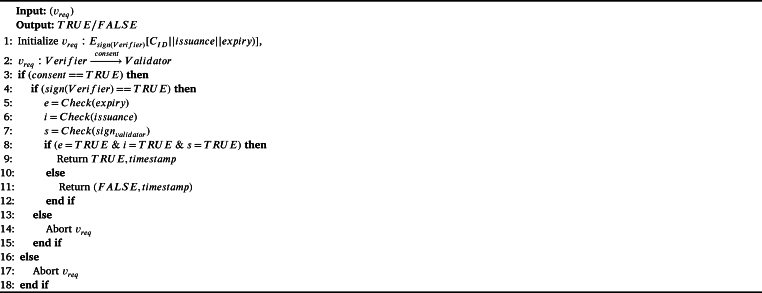
*CredentialVerification*().

#### 2.4.3 Proof of authority

We include Proof-of-Authority (PoA) in our proposed BRON due to certain advantages: i) PoA shows more throughput as compared to other existing consensus protocols, ii) PoA is that it is more energy-efficient compared to other consensus algorithms like Proof of Work (PoW) and Proof of Stake (PoS), iii) PoA is less computationally intensive, as it doesn't require miners to solve complex mathematical problems to validate transactions, iv) PoA does not require validators to be constantly monitoring their computers, and v) the stake of non-performing nodes is the reputation attached to the identity of the validators for which they do not want to lose their identity and always validating the transactions accurately. We summarize the operations of PoA for the BRON framework in Algorithm 3. All the authorities are considered to be the validators in the proposed BRON. When a transaction of verifiable credentials arrives in the blockchain, the validators are invoked to participate in PoA. We assume all the validators (a set of validators V) already have their corresponding registration keys (public and private key pair) following Algorithm 1.

**Algorithm 3:**
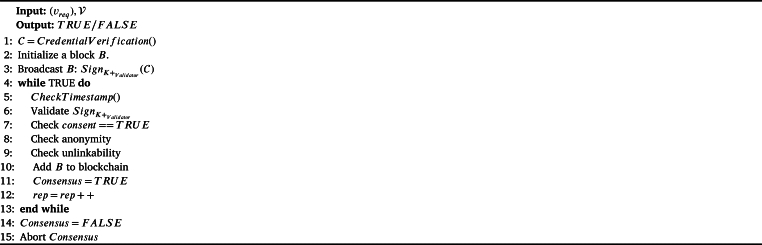
*PoA*().

#### 2.4.4 Smart contract

We show a smart contract that helps to incentivize the employees or performance management in a human resource management system. Our proposed smart contract is generic and includes the best practices of the organizations for performance management. These best practices include experience in terms of time spent in the organization and the basic notions of Key Performance Indicators (KPIs). We show the smart contract algorithm in Algorithm 4. In this algorithm, we consider *n* number of KPIs applicable in an organization. And we consider KPI() as a function for calculating the value of the KPI of an employee hang $EMP_{ID}$. Note that, the smart contract algorithm shown in Algorithm 4 is an example only, such smart contracts can be customized or modified according to the system requirements. We keep this scope open for future researchers and organizations to design and develop smart contracts applicable to real-life organizational environments. The benefit of such openness in the KPI calculation enhances the trust in the organization and intensifies employee-employer relationships.

**Algorithm 4:**
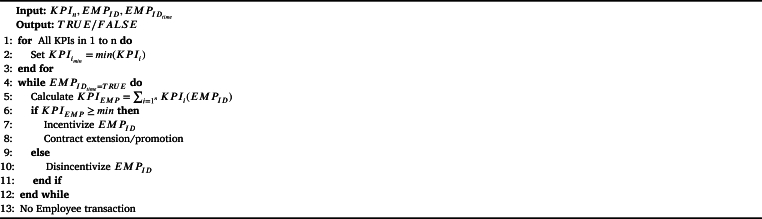
*SmartContract*().

#### 2.4.5 Privacy information retrieval

We use Privacy Information Retrieval (PIR) that guarantees differential privacy, which is a mathematical framework for ensuring the privacy of individuals in datasets. Differential privacy provides a strong guarantee of privacy by allowing data access without revealing sensitive information about any individual in the dataset. We assume the block transactions in our proposed BRON consist of sensitive and confidential information. We consider a query space $Q:q_{1},q_{2},...,q_{j}$. We define two privacy parameters *ϵ* and *δ* to denote privacy parameters for unlinkability and anonymity. Unlinkability defines that any data access is unlikable to another part of data in a block or other blocks; anonymity says that any part of data access should not reveal the identity of the owner or user. We define the privacy budget Ψ as in Equation (1).

**Figure 4:**
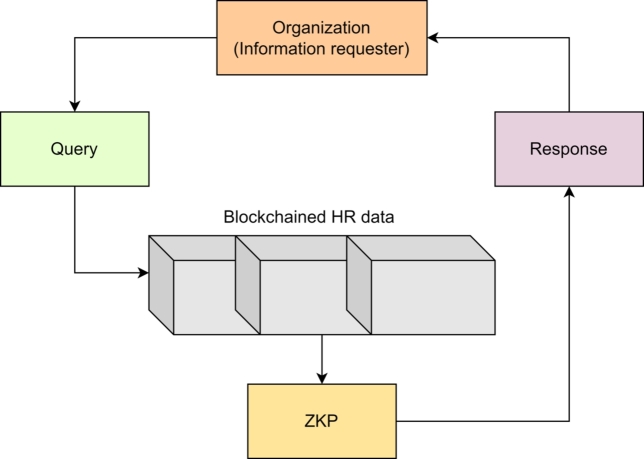
Flowchart for PIR in BRON.

(1) Ψ=cost(ϵ)+cost(δ),

where cost(ϵ) is calculated by the number of queries required to break unlinkability and cost(δ) is calculated by the number of queries required to break anonymity. We summarize the process of SPIR in Algorithm 5. It shows that the algorithm inputs *B*, *Q*, *ϵ*, *δ*, Ψ, and a Zero Knowledge Proof (ZKP) protocol. We use Zk-snark for ZKP [36]. The algorithm initializes ZKP with the required functions: i) GenWitness() function generates a witness (*w*) that is private, ii) TransCompCons() converts *w* into polynomial P, iii) CommitToPolynomials(polynomials) calculates commitment value, iv) CreateProof() generates proof of privacy, v) GetProofFromProver() is used to get the proof value from the receiver, and vi) VerifyProof() verifies proof with the inputs of verification key and the received proof from the receiver. If the verification result is true, SPIR calculates the noisy response $R_{i}$ with noise parameter *ρ*. If the verification result is false, we abort the PIR computation. Note that, to emphasize the consent management of the individuals, the PIR process checks the consent to be true, else the information retrieval process is a failure. Another aspect PIR includes is the permission to the requester of the data access with all the privileges of a data owner. This is checked by the requester's signature verification process. The query requester provides its signature. If the signature matches the signature of the blockchain data, the requester is the data owner, and the smart contract grants RADP(). For each query requested by the prover or requester, Algorithm 5 also checks the privacy budget Ψ to run the ZKP as shown in the steps from 5 to 8 in the algorithm. Indeed, ZKP is previously used for privacy preservation in blockchain; however, the use of ZKP in PIR with privacy budget analysis is novel for privacy preservation techniques. We show the logical diagram of PIR used in BRON in Fig. 4.

**Algorithm 5:**
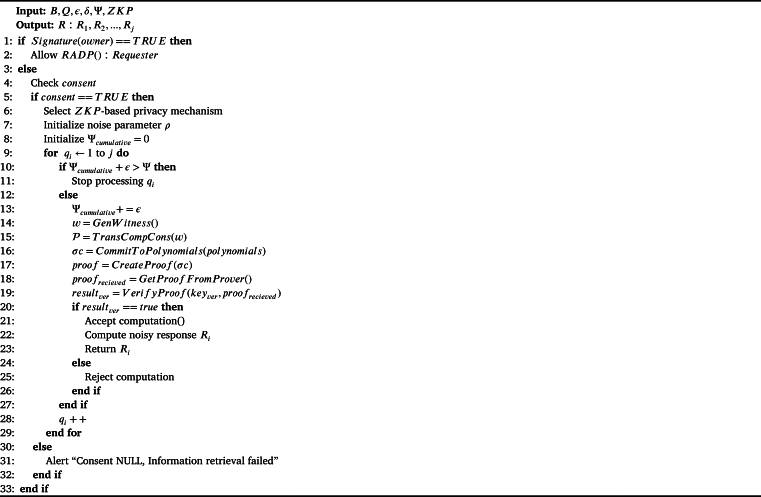
*PIR*().

## 3 Privacy analysis

In this section, we first discuss the threat model in Section 3.1 followed by an analysis of the privacy properties of our proposed BRON in Section 3.2. We emphasize three prime attributes of privacy: differential privacy, unlinkability, and anonymity.

### 3.1 Privacy threat model

Let A be an adversary. A sends *m* queries to link the obtained information and reveal the identity of the data owner. Therefore, the cost as shown in Equation 1 becomes *m* assuming that the attacker is successful in breaking the privacy. Note that, we are more interested in linking information together and not considering the confidentiality of the data, which is achievable through encryption techniques separately. We can formally execute the threat modeling with the following attributes.

• *Entities and Variables*: *Q* is the set of queries, A is an adversary, PR is a set of protective measures, *D* is the set of sensitive data in blockchain, and *T* is the set of time instances.
• *Threat scenario*(TS): Adversaries attempting to link multiple transactions and reveal the identity of the data source and strengthen the probability of further attacks on the revealed identity.
• *Security policies*: Cryptographic measures such as keys and ZKP. We assume that the cryptographic measures used in the proposed BRON are secure enough.
• *Probability distributions*: P(D) is the probability distribution over sensitive data, P(A) is the probability distribution over potential adversaries (in case of multiple adversaries), P(TS) is the probability of occurrence of unauthorized data access threat.
• *Risk assessment*: We can measure the risk based on Equation (2).
  (2) Risk(TS)=P(D)⁎P(A)⁎P(TS).

### 3.2 Attainment of privacy

As mentioned earlier we emphasize differential privacy, which is based on unlinkability and anonymity properties in the blockchain transactions or data.

*Confirming unlinkability*  Let the attacker send *m* queries for the blockchain data *D*. When the credential verification is in execution the VC is encrypted. The probability of revealing the VC without the key access is 0. In the worst case, the allowed data access only denotes the proof through ZKP, and PoA execution confirms the unlinkability. Thus, A is unable to deduce any link among the requested data access. Thus, unlinkability is assured in BRON. The cost of unlinkability becomes 0 which is optimum for an efficient privacy-ensured system.

*Confirming anonymity*  While registering the nodes (members), BRON provides public keys, which are the identities of the blockchain participants. Algorithm 1 shows that we generate the public keys *K*+ with hash operations on organization IDs; this means it is very unlikely to obtain the original identities of the organization or any nodes (members) from the BRON framework. Thus, the cost of anonymity becomes 0 which is optimum for an efficient privacy-ensured system.

*Differential privacy*  Differential privacy in blockchain connects unlinkability and anonymity by obscuring individual data points, enhancing confidentiality and user privacy. Differential privacy adds noise to data and prevents re-identification, where it ensures that transactions cannot be traced back to specific users. This in turn aligns with the principles of unlinkability and anonymity and enhances trust among the nodes. Therefore, we analyze differential privacy in BRON. We use a loss function based on the previously mentioned privacy parameters of *ϵ* and *δ* and their actual values. The formula is shown in Equation (3). Both the values of *ϵ* and *δ* are expected to be small enough to ensure string differential privacy [37].

(3) $$Loss(\epsilon_{actual},\delta_{actual})=|\epsilon_{actual}-\epsilon |+|\delta_{actual}-\delta |.$$

From the above discussion of unlinkability and anonymity, we put the values of $\epsilon_{actual}$ and $\delta_{actual}$ as 0. The system values of *ϵ* and *δ* should be 0. Thus, loss of privacy is 0, and we achieve differential privacy effectively in our proposed BRON.

## 4 Performance analysis

In this section, we first describe the implementation details in Section 4.1. We also mention the performance metrics and analyze the results based on the performance metrics in Section 4.2. We also do a comparative analysis of our proposed BRON to the existing models in Section 4.3.

### 4.1 Implementation

**Figure 5:**
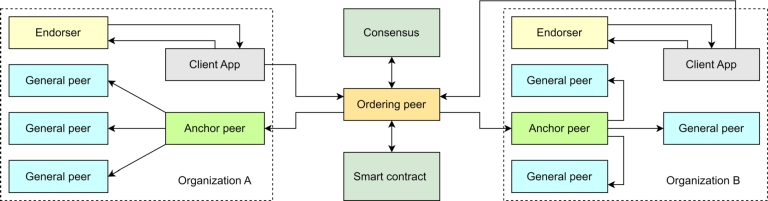
Network model used for experiments on BRON.

We use two servers as an organization consortium model for Hyperledger Fabric. The two servers are the Intel Xeon Gold processors with 8 cores, 3.8 GHz clock speed, and 3 TB memory extendable with SSDs. The peers, endorsers, and orderer (ordering peer) are the computing systems with 16GB RAM with Intel Core i7-7500U CPU @ 2.75 GHz and 2.90 GHz and 1 TB HDD. We show the network model in Fig. 5. We use the following steps to implement BRON. The detailed implementation details are available in [38] and [39].

• Download the platform binaries, including cryptogen.
• Initialize BYFN network and its additional flags as BRON uses CouchDB as the world state database.
• Define the network configuration in YAML format, including organizations, peers, orderers, channels, and policies.
• Being a consortium, we use the key generation method separately in Python and connect to hyperledger using Fabric SDK fabric−sdk−py.
• We change the profiles of the BYFN network consisting of two organizations. We also add three general peers, one endorser, and one anchor peer for each organization.
• We develop the chaincode in Java language. Package the chaincode into a chaincode package using the peer lifecycle chaincode package command.
• Create the identities.
• Create channels using the created channel configuration.
• Use Fabric Python SDK to join peers from each organization to the created channels.
• Initialize a connection profile to connect to the Fabric network using the Fabric Python SDK.
• Use the SDK APIs to submit transactions to the network, invoke chaincode functions, and interact with the ledger.
• We use Hyperledger Caliper to measure the performance of our proposed system.

*Evaluation metrics*  We measure the performance of BRON based on two basic parameters: throughput and latency as suggested by the Hyperledger Fabric Benchmark Performance metrics in Caliper. Both throughput and latency infer the knowledge about the scalability of the system. Low throughput and high latency say that the system is not scalable and high throughput and low latency say that the system is scalable; the increasing number of transactions do not degrade the performance at a large. We define the mentioned metrics as follows.

• *Latency*: We define latency as the time elapsed between a submitted read request and the received reply.
• *Throughput*: Transaction throughput represents the speed at which legitimate transactions are recorded and confirmed by the blockchain within a specified period.

We observe 10000 transactions of HR records and apply 100 queries. The obtained results are discussed in Section 4.2.

*Dataset*  We use the dataset for PIR to generate queries for different features of the dataset. The other functional parts as VC, PoA, and the smart contract do not need an explicit dataset; rather the execution of these functions generates a blockchain dataset. For PIR, we have explicitly used a hypothetical dataset consisting of 10000 transactions (rows). The dataset consists of various types of data including, the name of the employee, employee IDs, verification IDs (passport number/ national identity), sex, age, address, contact number, email id, bank account number, salary/payroll, KPI score, marital status, qualification credentials, department, position, pay rate, skillset, training details, attendance, previous two employments, health status, insurance amount, sexual orientation, and legal obligations. Overall we used 35 features in the dataset. We classify these features of the dataset in two parts: non-classified and classified. Classified features such as verification IDs, VCs, pay rate, address, and banking details are kept secret and need PIR for any further access. The other information related to employees is kept open in blockchain. For the generalization of our proposed approach and to check the applicability of BRON in the real-life applications of HRM, we also use the datasets available at: https://www.kaggle.com/datasets/rhuebner/human-resources-data-set?resource=download.

### 4.2 Results of BRON

**Figure 6:**
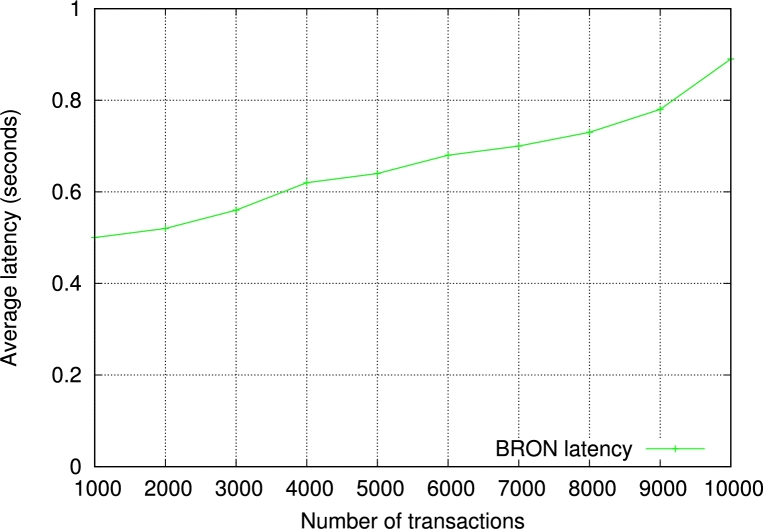
Observation of latency in the proposed BRON.

We measure the latency for transactions in BRON ranging from 1000 to 10000 transactions. The average latency is 0.662 seconds, which is almost the same for the traditional operations in hyperledger fabric. The increasing factor for latency is 0.041. The generalized latency cost with *n* number of transactions becomes O(0.1×logn). The experimental results of latency also suggest that BRON does not create any overhead in hyperledger fabric operations. We show the results in Fig. 6.

**Figure 7:**
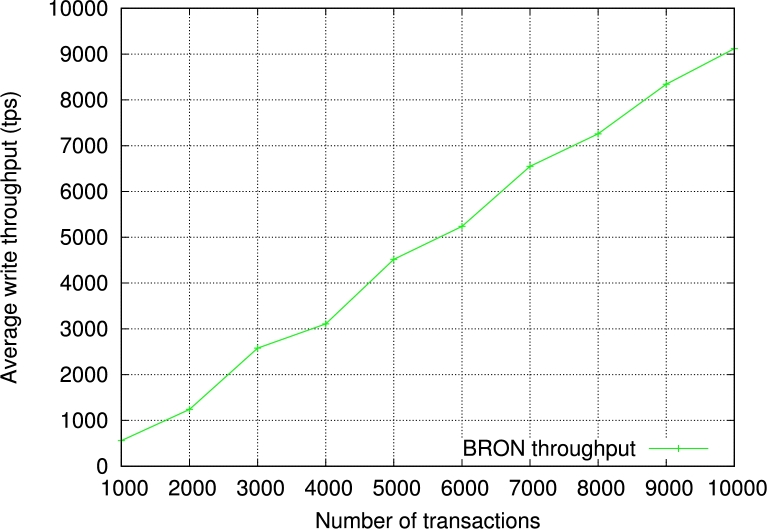
Observation of write throughput in the proposed BRON.

**Figure 8:**
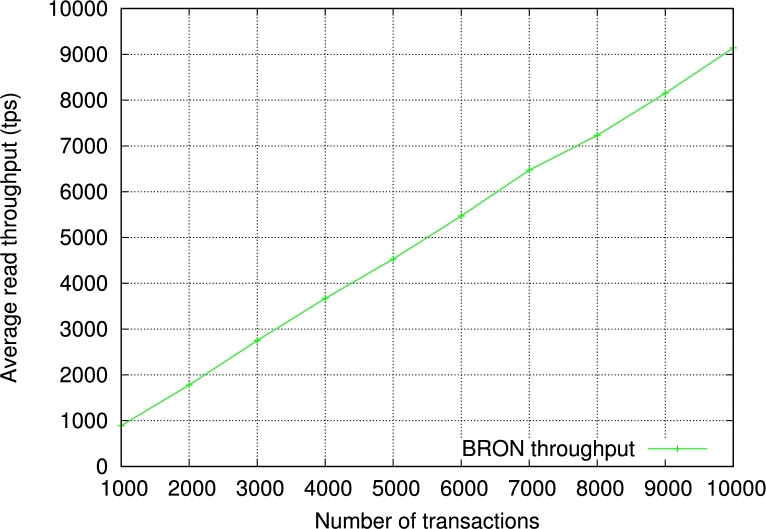
Observation of read throughput in the proposed BRON.

Next, we measure the throughput of BRON. We show the results based on input transactions which vary from 1000 to 10000. The average throughput of BRON is 4851.4 tps. The generalized throughput with *n* number of transactions becomes O(≡0.56n). We show the detailed results in Fig. 7. Please note that the term “transactions” considers only the transactions getting validated and saved in the blockchain. Furthermore, we have also experimented with BRON to measure the read throughput. We observe a linear incremental graph for the read latency as shown in Fig. 8. This helps in reading the transactions efficiently for the PIR and infers that the read operations in BRON are stable and do not provide any delay in the PIR process. As a result, the proposed PIR also becomes faster. Thus, BRON also ensures scalability as we see the increased write and read throughputs.

We perform a small experiment for PoA and PBFT to ensure that PoA is the best choice for our proposed BRON. We compare both the consensus mechanisms within the proposed BRON framework based on average throughput, average block finality time, and complexity. The average write throughputs of BRON using PoA and PBFT are ≈5000 tps and ≈3850 tps; it means that PoA is 29.87% better in throughput. The average block finality time for PoA is 25% less than the PBFT. PBFT requires $O(n^{2})$ messages to reach consensus, whereas PoA uses (logn) messages. The time complexity of PBFT is usually higher due to its multiple rounds. On the other hand, PoA is more precise as the nodes are limited in number and do not require extensive intra-node communication to reach consensus. All these benefits of PoA make a strong recommendation for selecting this consensus mechanism for our proposed BRON.

### 4.3 Comparative analysis

BRON is the first framework for the application of PIR in human resource management. The use of verifiable credentials is also new in this direction of the privacy information retrieval process. Therefore, we have chosen the candidate literature works, which show some significant contributions in the direction of privacy enhancements in human resource management systems: i) B. Goavarthi et al. [23], ii) T. H. Kim et al. [24], iii) Rahman et al. [28], iv) M. Damle et al. [30]. We show the comparative analysis in Table 2. In the table, we use *m* for members or nodes participating in the blockchain framework and *n* is the number of transactions.

**Table 2:** Comparative analysis.

Reference	Privacy methods	Anonymity	Unlinkability	Complexity
B. Goavarthi et al. [23]	Not applicable	No	No	*O*(*mn*)
T. H. Kim et al. [24]	Privacy level classification, encryption-based privacy attainment	Yes	Yes	*O*(*mlogn*)
Rahman et al. [28]	Not applicable	No	No	*O*(*logmn*)
M. Damle et al. [30]	Not applicable	No	No	*O*(*mlogn*)
Proposed BRON	PIR, ZKP, Encrypted VC	Yes	Yes	*O*(*mlogn*)

From the above comparative study, we claim that our proposed BRON is efficient for privacy-ensured blockchain transactions and information retrieval. Overall, BRON provides 30% reduced latency and 35% better throughput than the existing frameworks.

### 4.4 Remarks on applicability

In real-life scenarios, our proposed BRON can be used for multiple HR operations. The onboarding process can be smoother with verifiable credentials and a legitimate candidate can be onboard for a position. BRON can enhance the onboarding process reducing paperwork and streamlining new employee registration. Transparent performance evaluations on the blockchain enable fair and objective assessments of employee performance through the PIR in the proposed BRON. Employees' credentials and certifications are stored on the blockchain, enabling secure and efficient verification by recruiters and HR managers. Besides, the observed results of the BRON experimentations show that our proposed BRON is good in throughput and provides low latency. This performance applies to small-medium organizations connecting to the blockchain. For large companies with large multi-stakeholder entities, BRON may incur some latency; however, we can use a sharding technique or specific offline management operations to reduce the latency further. Overall, our proposed BRON is a significant solution for HRM decentralization.

### 4.5 Limitations of BRON

Blockchain frameworks inherently face complexity challenges and scalability issues. Though the complexity of the proposed BRON is not high, the scalability of the system may decrease in the long run. As a solution, we can apply some sharding techniques based on smart contract conditions. Apart from this, we can also use a data offloading mechanism to operate the blockchain in an offloaded mode for some operations to keep the online operations smooth. Apart from this, another challenge BRON may face is regarding the cryptographic techniques. One problem regarding the cryptographic techniques is the post-quantum security assurance. In such cases, we can use NIST-approved post-quantum secured algorithms rather than general cryptographic protocols. Another extension we aim to add in future is the transaction anonymity while updating transactions in the blockchain. For this, we can use blockchain mixing services or cryptographic techniques such as ZKPs or transaction aggregation schemes.

## 5 Conclusion

In this paper, we introduce BRON as a blockchain framework for human resource management decentralization. BRON, the first PIR-based framework for human resource records uses ZKP for privacy preservation and ensures anonymity and unlinkability. Besides, the use of verifiable credentials also provides benefits of one-time verification with expiration accordingly. Thus, BRON addresses the existing privacy problems of a decentralized human resource management framework. The key contributions of the proposed BRON are: i) verified credentials for ease up the data validation of the employees for the recruitment process, privacy-ensured data handling in blockchain by the organizations, and transparent mechanism of employee incentives. Overall, BRON becomes a suitable solution framework for HRM. From the point of performance, BRON shows good throughput and less latency. However, scalability can be an issue in the long run due to the increased number of employees and their records. Furthermore, in the future, we would like to include the self-sovereign identity for verifiable credentials to make the framework more generic.

## CRediT authorship contribution statement

**Gulshan Kumar:** Writing – review & editing, Writing – original draft, Formal analysis, Conceptualization. **Rahul Saha:** Writing – original draft, Validation, Methodology, Data curation, Conceptualization. **Manish Gupta:** Validation, Supervision, Project administration, Investigation, Data curation. **Tai Hoon Kim:** Validation, Supervision, Project administration, Investigation, Data curation.

## Declaration of Competing Interest

The authors declare that they have no known competing financial interests or personal relationships that could have appeared to influence the work reported in this paper.

## Data Availability

There is no specific data used from any organization or institution to make it publicly available explicitly. However, the use of programming code can be shared based on the required requests.
